# The strategic use of lecture recordings to facilitate an active and self-directed learning approach

**DOI:** 10.1186/s12909-016-0723-0

**Published:** 2016-08-12

**Authors:** Luminica Topale

**Affiliations:** School of Medicine, St. George’s University, St. George’s, Grenada

**Keywords:** Medical education, Lecture recordings, Strategic learning, Learning strategies, Active learning, Self-directed learning

## Abstract

**Background:**

New learning technologies have the capacity to dramatically impact how students go about learning and to facilitate an active, self-directed learning approach. In U. S. medical education, students encounter a large volume of content, which must be mastered at an accelerated pace. The added pressure to excel on the USMLE Step 1 licensing exam and competition for residency placements, require that students adopt an informed approach to the use of learning technologies so as to enhance rather than to detract from the learning process. The primary aim of this study was to gain a better understanding of how students were using recorded lectures in their learning and how their study habits have been influenced by the technology.

**Methods:**

Survey research was undertaken using a convenience sample. Students were asked to voluntarily participate in an electronic survey comprised of 27 closed ended, multiple choice questions, and one open ended item. The survey was designed to explore students’ perceptions of how recorded lectures affected their choices regarding class participation and impacted their learning and to gain an understanding of how recorded lectures facilitated a strategic, active learning process.

**Results:**

Findings revealed that recorded lectures had little influence on students’ choices to participate, and that the perceived benefits of integrating recorded lectures into study practices were related to their facilitation of and impact on efficient, active, and self-directed learning.

**Conclusions:**

This study was a useful investigation into how the availability of lecture capture technology influenced medical students’ study behaviors and how students were making valuable use of the technology as an active learning tool.

## Background

New e-learning technologies have become ubiquitous in educational settings as institutions strive to maintain technological currency and to offer students and faculty more effective educational tools. E-learning tools can facilitate an adaptive, self-directed approach to learning [1]. Nonetheless, the misuse of the technology could prove academically disadvantageous if it detracts from active learning, leads to an inefficient use of study time, or fosters study strategies that prevent the critical application of lecture content. In particular, pre-clinical students are under pressure to quickly master a large volume of course material and to develop the comprehensive knowledge and skills required to achieve a top score on the Step 1 U. S. medical licensing exam, and eventually a successful residency match.

Lecture capture technology is one of the most popular e-learning technologies currently available to medical students. It offers students the flexibility to review lectures anytime, anywhere, with the option of pausing the recording or adjusting the pace at which it is viewed. Furthermore, it enables faculty to provide their lecture audio, video, and Power Points. Nonetheless, there is widespread concern among teaching faculty that providing recorded lectures has a negative influence on medical students’ live lecture attendance [2–6].

While there is justification for the widespread use of e-learning technologies such as lecture capture [1] the extent to which it is embraced by students or leads to positive learning outcomes has yet to be presented. Tomoko et al. [7] offer evidence that lecture participation has been adversely affected by the availability of recorded lectures; however, a number of other studies have provided contrary evidence [2–5, 8, 9]. It is also unclear if medical students are cautious in their use of recorded lectures for learning [3, 10] or if they, in fact, use it extensively as a learning tool [1, 3, 4, 9]. Likewise, the impact of recorded lectures on learning outcomes is unclear. Some evidence suggests that use of recorded lectures results in better grades [7, 11, 12]. Other studies suggest that use of recorded lectures does not positively impact performance [4, 6, 13, 14] or may, in fact, be associated with poorer performance [6, 10, 15]. This may be contrary to what students perceive to be true [14]. Deriving from evidence that recorded lectures have become widely available and feature among the learning resources medical students turn to, recent studies have sought to explore how and why students make use of this technology [4, 5]. Nonetheless, there is little knowledge of how students make strategic use of recorded lectures, or of how to best integrate these technologies into the learning process.

Therefore, the aim of the current study was to further investigate how the availability of recorded lectures has influenced traditional lecture participation, what students perceive to be the advantages and disadvantages of integrating this technology into their learning approach, and how students are strategically using recorded lectures for learning. It was anticipated that the findings will be helpful to medical students, teaching faculty, and student academic support personnel in the development of a best practices approach to integrating recorded lectures into the learning process.

## Methods

In light of the conflicting and limited evidence on the impact and best use of recorded lectures in medical education settings, an institutional research project was undertaken at an international medical school with a fast-paced curriculum and a strong commitment to providing student academic support. Two years before this present study, the School of Medicine had made recorded lectures available to students via Mediasite’s Sonic Foundry lecture capture technology. Matriculating class intake numbers consistently exceed 500 students, making lecturing the primary teaching method; therefore, recorded lectures were offered to complement students’ learning outside the traditional lecture hall.

After a search of both the Buros Institute of Mental Measurements and the Educational Testing Service (ETS) databases failed to identify an instrument for the assessment of the implementation of technology into student learning at the tertiary level, the researcher designed an appropriate survey instrument. Recommended survey design principles were followed [16]. Items and options were designed to reflect existing themes in medical education literature exploring the use of recorded lectures as well as anecdotal findings from the author’s own experiences as a medical learning strategist.

The researcher piloted the survey with 28 assigned medial student advisees representing the two years of student cohorts. These advisees were selected because they had, during advising meetings, specifically enquired about the best use of recorded lectures. A link to the survey was sent in an email, which offered the advisees an opportunity to voluntarily take the survey, offer additional response options by way of an “other” category, and recommend additional items or ways in which the existing items could be improved.

The pilot enabled the researcher to experiment with the features of the survey design software (Angel) to identify its features and limitations, to test out the administration process, and to revise survey items. Input from the advisees informed survey revision. Analysis of this feedback revealed that some of the items choices were not exhaustive. Additionally, to more deeply investigate the use of recorded lectures for learning, three items asking about time spent on Mediasite relative to its value, perceived benefits, and perceived disadvantages were added.

The final survey comprised 27 closed ended, multiple choice questions, several with forced responses; and one open ended item. Sections included a) participant details, b) student profile, c) learning experiences prior to medical school matriculation, and d) learning experiences during the past three months of study (the current academic term). Requested participant profile details included gender, age, region of origin, academic term of study, and approximated grade point average (GPA). The prior learning experiences section was designed to gain an understanding of students’ lecture participation and use of recorded lectures prior to medical studies. The current learning experiences section examined lecture participation, the use of recorded lectures, the reasons for their use, the manner in which they are incorporated into studies, the extent to which participants felt this use had impacted their learning approach, and recommendations for effective use of the technology by others. The open-ended item encouraged students to describe the best manner in which to use recorded lectures for learning.

Institutional IRB approval was obtained from the institution in order to conduct the study. One month prior to examinations, all first and second year medical students were invited to participate in the electronic survey available on the institution’s learning management system. A convenience sample was used to gain insights from a wide cross-section of medical students. Invitations with a survey link were sent to students’ university e-mail accounts using the established e-mail groups for all registered year one and two students. To meet the requirement for informed consent, the invitation indicated that the survey aimed to gain a better understanding of how students were using recorded lectures in their learning and how their study habits have been influenced by the technology. Students were also informed that all submissions were voluntary and anonymous. No reward was offered for participation. The survey was open for three months; and subsequent to the second invitation, three reminders were sent at intervals during the submission period.

The learning management system’s survey feature collated individual responses. Analysis involved a calculation of response frequencies. This data was exported to Excel to facilitate the reporting process. In order to determine how representative the participants were of the entire population of enrolled students, demographic data was compared to student registration records provided by the Registrar’s Office.

## Results

### Participant profile

There were 281responses from the 2,322 surveys emailed using the institution’s established medical student email address groups. As illustrated in Table 1, the majority of participants were North American (85.5 %) and relatively split between Year 1 (49.1 %) and Year 2 (50.4 %), with representation from all four academic terms (T1-24.5 %, T2-24.6 %, T3/4-23.4 %, T5-27.5 %). Participant GPAs below 2.5 (15.6 %), 2.5–2.9 (23.4 %), 3.0–3.5 (31.9 %) and 3.5–3.75 (11.3 %) were also noted. The age of participants covered a variety of ranges: 20–24 (34.7 %), 25–29 (53.5), 30–34 (8.6 %), and 35+ (3.3 %). The demographic data of the participants with respect to age, region of origin, year of study, and GPA were reflective of institutional enrollment data with the only exceptions being approximately 11 % greater participation among the 20–24 age group (34.7 %), approximately 13 % less representation from the 25–29 age group (53.5), and approximately 9 % more representation for students with GPAs of 3.75–4.0 (13.8 %). Variations between participant and institutional data were less than 5 % for all other demographic categories.

**Table 1:** Participant profile comparison with total school of medicine population student demographic profile

		SOM	Respondents	Variation
Total Number		2322.0	281.0	
Origin	Africa	2.7	1.4	1.3
	Asia	4.3	1.4	2.9
	Australia	0.0	0.0	0.0
	Europe	2.8	2.1	0.7
	North America	85.4	87.5	−2.1
	South America	4.5	11.7	−7.3
	Unspecified	0.2	0.0	0.2
Academic Term	Term 1	24.5	29.4	−4.9
	Term 2	24.6	22.6	2.1
	Term 3	0.5	2.2	−1.7
	Term 4	22.9	23.3	−0.4
	Term 5	27.5	22.6	4.9
Age	20–24	34.7	45.9	−11.2
	25–29	53.5	40.2	13.3
	30–34	8.6	9.3	−0.7
	35–39	2.2	3.9	−1.7
	40+	1.1	0.7	0.4
GPA Range	<2.0	4.0	0.7	3.3
	2.0–2.4	15.6	11.1	4.5
	2.5–2.9	23.4	20.0	3.4
	3.0–3.4	31.9	30.0	1.9
	3.5–3.74	11.3	16.1	−4.7
	3.75–4	13.8	23.2	−9.4

### Class participation

As highlighted in Table 2, prior to medical school matriculation, 88.3 % of students responding to the survey attended lectures regularly or never missed a class, 7.8 % had inconsistent attendance, and 3.7 % only rarely or occasionally attended lectures. During the academic term of the study, 68.3 % of students indicated regular lecture attendance, 16.4 % reported inconsistent attendance, and 15.3 % reporting rare or occasional attendance.

**Table 2:** Percentage of responses for prior and current class participation

Attendance	Prior	Current
Excellent: I tried my best to never miss lectures	53.4 %	35.6 %
Very good: I attended fairly regularly	34.9 %	32.7 %
Inconsistent: I attended when I felt like it or if the topic was important	7.8 %	16.4 %
Occasional: I attended lectures once in a while	2.5 %	5.3 %
Rare: I generally did not attend lectures	1.4 %	10.0 %

Reasons for non-participation at lectures and strategies form improvement are presented in Table 3. Nearly 88 % of the participants reported that their attendance had not been negatively influenced by the availability of recorded lectures. Choices to attend live lectures depended primarily on the communication skills of the lecturer (30.9 %), the perceived value of attending lecture over reading notes and texts (18 %), and how engaging past lectures had been (14.7 %), with only 12.9 % reporting the availability of recordings as their primary reason missing lectures. When asked what three strategies would improve their participation (see Table 4), the most selected options were points for attendance (52.3 %), question and answer or quizzing by the lecturer (41.6 %), modeling of critical thinking by lecturers (40.2)%, and interactive lecture formats such as discussion (28.8 %). Interestingly, nearly 28 % indicated that nothing would improve attendance because they rarely miss lectures.

**Table 3:** Reasons for non-participation

Reason for non-participation	% of respondents
I have already studied the content in a previous course.	4.7
I can get the same thing from reading slides and texts.	18.0
I find the lecturer is difficult to understand.	30.9
I don't find the lecture engaging.	14.7
I can just watch the recording instead.	12.9
I do not miss lectures.	18.7

**Table 4:** Strategies that would improve participation

What would improve your class lecture attendance?	% of respondents
Bonus points for attendance	52.3
More Q&A/quizzing by the lecturers	41.6
More modeling of critical thinking by lecturers	40.2
More interactive lectures (ex. Discussion)	28.8
Nothing. I already try my best to never miss lecture.	27.8
Recorded attendance	17.1
Nothing. I can't think of anything that would make lectures useful to me.	13.5
Pop quizzes	12.1
Lecture notes not being provided prior to lecture.	4.6

### Strategic use of recorded lectures

How frequently respondents made use of recorded lectures (Table 5) and the reasons why students were using recordings (Table 6) were explored. Sixty-five percent of respondents reported using recordings on a weekly basis, with 39 % accessing recordings more than three times per week. Approximately 6 % used them primarily before major assessments, and 39.2 % rarely or never used recordings. Primary reasons for using the technology included making up for a lecture they had to miss (58.7 %), reviewing and clarifying lecture content following lecture attendance (55.9 %), developing or enhancing notes (44.1 %), and preparing for a quiz or exam (43.8 %).

**Table 5:** Reasons for using recorded lectures

What have been your primary reasons for using recorded lectures?	% of respondents
I do not use recorded lectures.	5.7
If I had to miss a lecture	58.7
Regularly instead of attending lectures.	22.4
To clarify/review material after attending a lecture	55.9
To develop/enhance my study notes	44.1
To review/clarify material for a quiz or exam	43.8
No clear pattern or reason for using recordings	2.5

**Table 6:** How frequently respondents used recorded lectures

Frequency of Use	% of respondents
I do not use recordings	5.0
More than 3 times per week.	39.1
Once or twice per week.	26.0
Just before exams or quizzes.	5.7
I rarely use recordings	24.2

Of those students who reported using recordings in a strategic way (Table 7), the majority used them in an active way—pausing recordings to take notes or to consult texts, lecture notes, or the Internet (44.8 %). Watching recordings once while making notes (21 %) and viewing only parts they felt they needed to review (15.7 %) were considered to be somewhat less active strategies. Passive approaches—watching lectures repeatedly (5 %) or watching them without taking notes (0.7 %) were the least favored strategies.

**Table 7:** Manner in which recorded lectures are used

How would you characterize your use of recorded lectures?	% of respondents
I do not use recorded lectures.	7.5
Watch the entire lecture once and listen but do not take notes	4.3
Watch the lecture once and take notes as it plays	21.0
Watch the parts I need to review and skip through the less important parts	15.7
Watch it, pause as needed to make notes, etc., then watch the next part	44.8
Watch lectures repeatedly to learn and review material	0.7
No system or pattern for using recorded lectures.	5.7

### Impact on learning

The effect of using recorded lectures was also explored. Table 8 highlights the respondents’ perceptions of how their performance was affected by their use of recordings. While over 28 % of the group felt that use of recordings significantly improved their performance, 58 % felt there was little or no positive impact on their grades. Few participants felt their grades had dropped as a result of using recordings (1.1 %).

**Table 8:** Impact of use on performance

To what extend has your use of recorded lectures affected your grade?	% of respondents
I don't use recorded lectures.	11.7
Quite a bit. Use of recordings helped to improve my grade	28.8
Somewhat. Use of recordings has improved my grades a bit.	23.5
Not much. I don't think my use of recordings made any difference.	34.5
My grades have dropped a bit because of my use.	0.4
My grades have dropped a lot because of my use.	0.7

Tables 9 presents perceived advantages and disadvantages of using the technology for learning. Among the greatest benefits of using recorded lectures were the simultaneous use of multiple modes of learning (27.4 %), an enhanced understanding of lecturers and content (27 %), control over pacing (17.8 %), and scheduling of learning activities (17.8 %). Only 2.1 % of the 281 respondents chose not having to attend class as the greatest benefit. Top disadvantages associated with recorded lecture use were primarily related to the technology: incomplete recordings (27.8 %), the unreliability of access (25.5 %). Other disadvantages reported include and the risk of spending too much time viewing recordings (22.4 %), falling behind (5.6 %), or identifying the best strategies for use of the technology (3.9 %). Few students (6 %) felt use resulted in truancy.

**Table 9:** Advantages and disadvantages

Which of the following is the greatest advantage/disadvantage of using recorded lectures?	
Advantages	% of respondents
I don't have to attend class.	2.1
It gives me flexibility to view lectures when convenient	17.8
It improves my ability to understand lectures and lecture content	27.0
It enables me to view lectures at my own pace.	17.8
It enables me to view lectures, consult texts and other resources, etc. at the same time.	27.4
I don't use it.	7.8
Disadvantages	% of respondents
It takes a lot of time to figure out the best way to use it.	3.9
It is easy to waste too much study time using it.	22.4
Recordings are incomplete (missing slides).	27.8
The technology is unreliable.	25.3
It leads to skipping class.	5.7
I don't use it.	9.3
It leads to falling behind.	5.6

The final survey item asked respondents to consider what advice they would give to new students thinking of using recordings in their studies. As Table 10 illustrates, 28.8 % of respondents recommended that new students should make extensive use of recordings while studying or reviewing. A large percentage of the respondents advised new students that recordings should be used only when necessary to clarify lecture material or prepare for exams (41.3 %) or to make up for a lecture they had to miss (38.1 %). Few students discouraged its use (4.6 %) or even encouraged it be used instead of attending lectures (10 %).

**Table 10:** Advice to New Students

What advice would you give to new students thinking of using recorded lectures for their studies?	
Advice to New Students	% of respondents
Avoid using it. It takes time and isn't necessarily helpful.	4.6
Only use it if you have had to miss a class.	38.1
Use it only when necessary before exams or to clarify class material.	41.3
Use it as much as possible when you are studying or reviewing.	28.8
Use it instead of attending lectures.	10.0

## Discussion

There is value in understanding if new learning technologies serve to complement or replace traditional learning activities. One of the aims of the present study was to identify how year one and year two medical students’ participation in lectures had been influenced by the availability of recorded lectures. While there were reported differences when medical school lecture participation was compared to pre-matriculation lecture participation, only 13 % reported the availability of recorded lectures as their reason for non-participation; likewise, nearly 30 % indicated that they tried their best to never miss lectures, with over 68 % attending regularly. This supports earlier findings that the availability of recorded lectures does not influence attendance [2, 9], with the majority of students continuing to attend [8].

### Class participation

Research suggests that factors other than the availability of recorded lectures negatively influence medical students’ choices to participate in live lectures [2, 9]. Participation appears to be determined by such factors as prior negative experiences with the lecturer, anticipated benefits of attendance, lecture quality and likelihood to engage the student, and learning preferences [2]. The effectiveness of the lecturer (organization, knowledge, preparedness, and delivery) further influences attendance [5]. Furthermore, students reported control over time, pacing, and learning methods as primary reasons for replacing class time with independent learning [3].

Supporting earlier findings, the current study identified the quality of the lecture (lecturer’s ability to communicate and engagement level) as the primary cause of missed lectures rather than the availability of recordings. Additionally, students considered the perceived learning gains associated with attending over achieving the same effect through independent learning activities. Although attendance points were a desirable lure to over 50 % of respondents, over 40 % indicated that a more Socratic approach or modeling of critical thinking by lecturers would improve their lecture participation. In light of this information, medical school faculty should consider the extent to which they engage their students in lecture by assessing their ability to apply lecture content, and in turn, modeling the cognitive processes involved in applying lecture content to clinical contexts.

The participants in this study demonstrated that although recorded lectures were embraced by most, a significant percentage chose to maintain more traditional approaches to learning, with 30 % reporting rarely or never using recorded lectures. McNulty et al. [10] reported that when recorded lectures were made available to medical students, over 50 % used them only sparingly; Pilarski et al. [9] found that 43.3 % of students used them frequently or very frequently; and Franklin et al. [4] found that over 80 % of year one and two students were regularly using them as a learning resource. Of the respondents in the present study, 65 % used them weekly, with nearly 40 % of this group accessing them more than three times per week, and less than one-third rarely or never using recordings. This evidence suggests an upward trend in the use of recorded lectures for learning.

### Strategic use of recorded lectures

McNamara [17] warns against a memorization driven *learning by consumption* model not uncommon in medical education, and encourages students and educators to adopt models and strategies that are active and result in deeper understanding of course material. Unless lecturers adopt strategies that engage students, viewing lectures, whether live or recorded, can be a generally passive process typical of strategies that develop only a surface understanding of course content [18]. Therefore, ineffective use of recorded lectures may result in increased task redundancy and passive learning [19, 20].

Alternatively, the strategic use of recorded lectures makes them a valuable resource in the development of self-directed learning in medical school [5]. The results of the present survey indicated that students where deliberate in their reasons for using recorded lectures for learning. Review and clarification of material following live lecture attendance and prior to assessments, development of study notes, and replacement for a lecture if one had to be missed were the most frequently reported reasons for choosing to access recorded lectures. The frequently reported reasons for using recorded lectures match the advice respondents gave to new users: clarification following lecture, preparation for exams, and replacement for a missed class.

Students who reported using recorded lectures in a strategic manner did so by pausing recordings to take notes or to consult other resources. A very small group (10 %) used them passively, either watching them once or repeatedly without any other learning activity. This suggests that the majority of the medical students in the study, were mature and discriminating consumers of lectures and purposeful in their use of lecture capture technology for strategic learning and supports earlier findings [2–4, 9].

Some research indicates medical students do not find recorded lectures to be especially useful as study aids [14]. Nonetheless, those who make use of the technology have reported finding it helpful when learning and reviewing [9]. Students have also reported better stress management, accelerated learning, more active learning, improved focus, and increased knowledge acquisition as the benefits of using recorded lectures [3, 9]. The active learning process associated with using recorded lectures for learning is one of the main benefits identified by students, along with the ability to pace and manage the learning experience while reviewing material [3, 4]. Beyond the benefit of managing how content is presented and perceived, students are also able to review and clarify content that they find particularly challenging, a stated benefit of e-learning technologies [1]. The present study further investigated this aspect of the technology’s impact by asking respondents to identify what they felt were the advantages and disadvantages of using recorded lectures for learning. Major benefits reported included facilitation of active learning, improved understanding of lectures, and enhanced time management. These uses and advantages support earlier research by Cardall and colleagues [3] and Franklin and colleagues [4].

Similar to the findings of Callas et al. [21] perceived disadvantages were primarily related to unreliable technology or inefficient use of the tool. The availability of recorded lectures may reduce medical students’ anxiety [9]; however, ineffective use of recorded lectures can lead to inefficiency [20] resulting in increased levels of time-related anxiety [22, 23]. More than one-quarter of respondents also indicated that the risk of spending too much time listening to lectures and experimenting with the best way to use the technology in the learning process was a notable disadvantage. This awareness of the potential disadvantages of the technology is reflected in the advice respondents offered new users of recorded lectures. Less than 30 % encouraged its extensive use, while approximately 40 % recommended limiting its use to making up for missed lectures, and the same proportion recommended it be used only when necessary to clarify class material or review for exams.

### Impact of recorded lectures on student learning outcomes

Determining the impact of recorded lectures on students’ performance has been challenging [9]. In veterinary medicine, increased viewing of recorded lectures was associated with better performance in basic sciences courses that relied most heavily on teaching via lectures [11]. In the context of medical education, perceived advantages to using recordings include gaining personal time, increased study time, and the ability to pause recordings to take notes [3]. Despite student perceptions, frequent reliance on recorded lectures for studying has indicated an adverse effect on exam performance [15]. McNulty et al. [10] also found that those who made frequent use of recorded lectures had significantly lower exam scores. One study did not find a statistically significant difference in performance between those who attended live lectures and those who viewed only digital lectures [13]. One study, which focused on students in podiatric medicine found significant learning gains, particularly for non-native English language speakers when a lecture-capture supported course was offered [12].

Recent studies indicate that frequent use of lecture recordings results in real performance gains [7] as well as perceived gains [3]. Although 29 % of respondents in the current study felt their performance improved as a result of recorded lecture use, 60 % of students felt use had little or no positive impact on performance. This is consistent with other findings where medical students did not especially value the use of recorded lectures in their learning process [14] and rated live lectures more favorably than the recorded version of the same lectures [8, 24]. Earlier findings suggest that although there is no difference in performance between those who attend lectures versus those who do not attend [6] or only view recordings [3, 13], frequent use of recordings for studying may in fact adversely affect performance [10, 15, 25].

Although grades were not formally analyzed in the present study, the majority of respondents who made use of recordings did not feel they had an impact on performance, while less than 30 % felt that they did. Earlier research suggests that medical students may not fully understand the impact of learning technologies on their performance [14]; nonetheless, analyses of performance data do not paint a clearer picture of the positive or negative impact on performance [6, 7, 10–12, 15]. Some research suggests there is no impact of recorded lecture use on performance [4, 6, 13, 14].

The link between strategic learning and student performance has been investigated by others. Research supports the argument that students change their learning strategies in response to the learning environment, the workload they encounter, and the methods by which they are assessed [26–28]. Management of effort and an organized approach to studying are related to this strategic approach [29] as are the drive to achieve, perform well on exams, compete with peers, and impress faculty—traits typical of medical students’ [26, 27]. Furthermore, medical students have the skill to vary their approach—surface or deep—depending on the task and expectations for achievement, thus performing better than those students who use a single approach [27].

The lack of consistent evidence implies that the relationship between the use of recorded lectures and performance may not be a direct one and more a product of the benefits and disadvantages of the use of recorded lectures for learning, among other learning resources. Students who are effectively strategic may benefit more than those who use recordings passively. Responses to the present study indicate that students felt their learning was more efficient, had better control over their rate of learning and their time, and were able to integrate multiple modes of learning into the process. These findings are supported by comments such as “I use [recordings] as an interactive, engaging method to review course material at [my] own pace, consulting texts, notes, atlas as needed to help understand the material” and “[Recordings] enable me to structure my day much more effectively, and coordinate my diurnal rhythms with study activities in a way that dramatically improves my learning.” Nonetheless, respondents were aware that it was possible to spend too much time viewing recording, and although nearly one-third of the group recommended that new students should actively use the technology, over 40 % advised that this use be very purposeful. Comments such as “a powerful tool, only if used wisely by students,” and “in the hands of procrastinating medical students…it has served as a tool of inefficiency” further suggest that students feel the need to be strategic when using lecture capture technology for learning.

### Limitations

This study aimed to obtain data on the impact of recorded lectures on learning from a wide cross-section of the medical students at the institution. Limitations of the present study include the submission of an anonymous survey instrument for gathering data and the low response rate. The reliability of any self-report survey is dependant on the honesty of the respondent. Nonetheless participants may have been those most interested in using recorded lectures for learning. Furthermore, institutional emphasis on live lectures may have biased students toward responses that valued lectures and downplayed the use of recordings. The limited response rate in this study can be blamed on a number of causes. A technical failure prevented students from accessing the survey on the initial notification. This may have discouraged students from attempting the survey after the second notification. The technical failure also drove the submission time into conflict with end of term course and instructor evaluations, a major examination period, and summer holidays. Students may have been more apt to participate had a reward been offered or the survey conducted in class using an audience response system.

Although anonymity encourages forthright responses, follow-up in the event of a low response becomes a problem, as does triangulation [30]. However, despite the low participation rate, findings do reflect those of earlier medical education studies on the influence of recorded lectures on participation and learning and the researcher's own observations. Additionally, the demographic distribution of participants by age, gender, country of origin, year of study, and GPA reflect the institution’s Registrar’s data. Nonetheless, a follow up study should be undertaken to validate present findings and to further explore the relationship between specific strategies for using recorded lectures and student performance. This study should be conducted during a lull in the regular academic term rather than during a peak study period or during the end of term.

## Conclusions

The current study aimed to add to the research on the use of recorded lectures by medical students by further investigating how the availability of recorded lectures has influenced traditional lecture participation and what students perceive to be the advantages and disadvantages of integrating this technology into their learning approach. It also sought to shed light on how students are strategically using recorded lectures for learning. Despite the belief that student attendance is negatively influenced by the availability of recorded lectures, findings from the current study suggest that medical students are mature and discriminating consumers of lectures, purposeful in their use of recorded lectures for strategic learning. Results revealed that the majority of respondents continued to attend lectures regularly, not influenced by the availability of recorded lectures when making their attendance choices, and that recordings are being used to enhance their understanding of lecture content. The findings also suggest that the majority of respondents in the study strategically used recorded lectures to complement lectures and felt they were an efficient educational resource capable of enhancing the active learning process. The flexibility of managing the time and pace at which lectures may be reviewed, and the ability to integrate a variety of learning resources into the review of recorded lectures content may be the most desirable features of lecture capture technology.

The findings do, however, also suggest that traditional didactic lectures may no longer appeal to the current generation of medical students who favor more active and engaging learning environments. Lecture attendance is strongly influenced by the quality and level of engagement of the lecture, suggesting that current medical students want to be more actively involved in the teaching-learning process. In fact, the method of content delivery may matter less than the active process used to learn [31]. Educators are encouraged to consider ways in which to make their lectures more active through discussion, demonstrations, and problem solving activities [17, 18].

Liaison Committee for Medical Education (LCME) standards encourage institutions not only to provide academic support and advising for students, but also to facilitate an active self-directed approach to the learning of medicine. Findings from the present study suggest that when recorded lectures are used in an active, self-directed way to enhance the learning processes, students experience benefits from using the technology. Students who have experience with effective learning strategies may serve as peer mentors for less experienced students, thus contributing to the development of a best practices approach to the use of recorded lectures for the active, self-directed learning of medicine. Nonetheless, there is need for more research into the relationship between the strategic use of recorded lectures for learning and academic performance before a best practices model to be used in student advising can be developed.
